# Maternity Service in Bristol

**Published:** 1952-01

**Authors:** G. Gordon Lennon

**Affiliations:** Professor of Obstetrics and Gynaecology, Bristol University


### MATERNITY SERVICE IN BRISTOL

### SOME ASPECTS OF INTEGRATION AND POSSIBLE DEVELOPMENT

BY

G. GORDON LENNON, Ch.M., M.R.C.O.G., M.M.S.A.

*Professor of Obstetrics and Gynaecology, Bristol University*

> " *The problem is not simply whether midwifery should be in the hands of doctors, midwives, or specialists, or whether confinement should be at home or in hospital, but what will give the best results from the point of view of the patient and community at large.*"
>
> (D. Baird, 1951)

I have quoted the above sentence from an article on " Organization of Obstetric Service " because I think it expresses a principle that we may be inclined to forget. My suggestions for integrating the Obstetrical services in Bristol are based on closer teamwork between hospital, local authority clinics, general practitioners and midwives. In the past, perhaps, each aspect of the service has been too concerned with its own protection and too little with the larger aspect of co-operation and integration. A justifiable argument would be that closer teamwork would lead to *supervision* of all branches of the work, a term which may be disliked but which is necessary in any comprehensive outlook on the service.

### HOME AND HOSPITAL CO-OPERATION

I believe I can incorporate general practitioners and midwives into the Maternity Service by encouraging them in the view that they are part of a team consisting of consultant and themselves. The individual practitioner can work with the midwife in his district and also as clinical assistant to the consultant of his choice, not only by referring those cases which he desires the consultant to see but by filtering all his cases through the consultant clinic at least twice, early in the ante-natal period and again at the thirty-sixth week, even if he is delivering the patient in her own home or in a General Practitioner Hospital or Home. In other cases the consultant may reciprocate by getting the general practitioner to supervise the ante-natal care of some of his cases that are eventually to be delivered in hospital for medical or social reasons. The consultants may not like this because it would mean more work for them, but with the facilities available to them in the form of Registrars this would largely be a matter of delegation of responsibility. Its advantages are that patients would have contact with hospital, and practitioners would have done for them blood tests and ancillary examinations, for example, X-ray of the patient's chest if required. Another advantage would be that a few patients could be booked for hospital at an earlier date due to the discovery of an obstetrical indication which might have escaped the general practitioner. The hospital would thus be *supervising* (if I may use the term)

those patients booked for hospital and those for domiciliary confinement. Should anything go wrong during domiciliary confinement the hospital specialists would not be strangers either to doctor, midwife, or patient. In the same way the general practitioner could " supervise " midwives' cases and midwives would have the benefit of the practitioner's opinion which might obviate some visits to hospital clinics.

*Local Authority Clinics.* The number of Local Authority Clinics in Bristol is too great. The consultant in many cases is wasting time attending short sessions at outlying clinics. I feel that there is need to gather together many of these clinics and have them run by doctors particularly interested in obstetrical work who would be regarded as clinical assistants to the hospital department. This would make the educational aspect of ante-natal care easier to deal with and case-notes more easily available. It would cut down the number of clinics, which would also mean that the doctor would have time to come and see cases in hospital and perhaps take part in relieving the work of the hospital unit. He (or she) would not be entirely an " ante-natalist ".

I can visualize then that there would be a group of consultants responsible for a number of clinics and having a number of clinical assistants under their jurisdiction and also a number of general practitioners and midwives.

*Emergency Obstetric Service.* There is need in Bristol for the better treatment of cases of haemorrhage and toxaemia of pregnancy. Dr. A. E. Ross (1951) has shown in a study of 172 maternal deaths occurring in 93,371 deliveries in Bristol between 1937 and 1949 that eclampsia had the highest number of preventable deaths—20 out of 35. Of 25 deaths ascribed to postpartum haemorrhage 7 were thought to have been preventable. The comparison of the figures of maternal deaths due to haemorrhage and toxaemia in Bristol with the rest of England and Wales is as follows:

| | Bristol (1937–49) | | England and Wales (1937–48) | |
|---|---|---|---|---|
| | No. | Percentage | No. | Percentage |
| Haemorrhage of pregnancy. Haemorrhage of Childbirth and Puerperium. | 32 | 20·3 | 2,795 | 15·0 |
| Toxaemia of Pregnancy. Puerperal Toxaemias. | 50 | 31·6 | 4,585 | 24·5 |

No greater arguments are needed than those above (if any argument is indeed necessary) for an efficient emergency Obstetric Service (" Flying Squad ") in Bristol.

*Other Services.* I feel that there is great need for co-ordination of the services closely associated with the maternity service, for example, Family Planning, Marriage Guidance, and Eugenics. Some integration of Mother and Child Welfare Clinics is necessary too. Perhaps the association of the post-natal clinic with the Mother and Child Welfare Clinic would be helpful. A mother will come to a clinic for her baby more easily than for herself. Her post-natal examination at the same time as the baby's examination at the Welfare Clinic may solve the problem of poor attendances at post-natal clinics.

### THE FUTURE

I believe that in the distant future there will be no such thing as domiciliary midwifery and that the hospital obstetrical department will have general practitioner beds and also consultant beds. (There is little or no domiciliary midwifery in Australia and New Zealand.) The improved figures of maternal and infant mortality rates are probably not unconnected with the increased trend towards hospital confinement. The so-called supervision would be much more harmonious if general practitioners and midwives did their work in hospitals and this would bring the two groups into still closer contact. Thus, I feel that groups of general practitioners will be responsible for the midwifery of the areas rather than individual doctors doing a few cases each. This may raise the criticism that each doctor is qualified to do maternity work. But similarly each doctor has a qualification to do paediatrics, and medicine, and surgery, and it would not be too much to suppose that some doctors, having interests other than obstetrical, would be paediatric general practitioners or medical general practitioners, etc. Perhaps the loudest blast with regard to this would naturally come from the midwives staffing the general practitioners' beds attached to the maternity unit. Such a scheme would lead the midwives to be regarded as nurses specially trained in maternity work, just the same as there are nurses specially trained in fevers and tuberculosis, and if we remember that " the problem is not simply whether midwifery should be in the hands of doctors, midwives or specialists or whether confinement should be at home or in hospital but what gives the best results from the point of view of the patient and the community at large ", then the counterblast should be ineffective!

Bristol has already seized the initiative in regard to a Maternity Service with the scheme proposed by the Local Medical Committee (1950) and I believe that with the co-operation of all interested parties there is real opportunity for Bristol to lead the country in this matter.

### REFERENCES

Baird, D. (1951). *Health Bulletin* (Department of Health for Scotland) 9, 66.

Ross, A. E. (1951) *Med. Off.*, 86 23.

Woolley, W. (1950) *Brit. Med. J.* ii (Supplement) 126.

